# Addressing the gender-specific barriers to cataract surgical services for women: evidence from Nepal

**Published:** 2022-12-16

**Authors:** Reeta Gurung, Radhika Upreti Oli

**Affiliations:** 1 Chief Executive Officer: Tilganga Institute of Ophthalmology, Kathmandu, Nepal.; 2 Senior Research Officer: Tilganga Institute of Ophthalmology, Kathmandu, Nepal.

**Implementing gender-responsive strategies and reducing the cost of eye care are necessary to improve women’s access to eye care services.**

**Figure:**
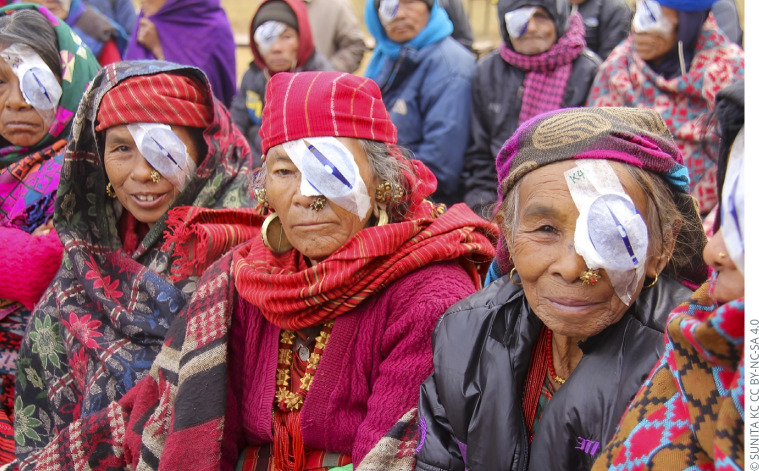
Women in Nepal bear a greater burden of blindness than men, but are less able to access the eye care they need. **NEPAL**

The burden of vision impairment and blindness is borne disproportionately by women around the world.^1^ In Nepal, the age-adjusted prevalence of bilateral blindness (presenting visual acuity <3/60 in the better eye) is 2.4% in women and 2.1% in men.^2^ Despite this, fewer women than men come to eye hospitals; they are more likely to visit rural outreach clinics where services are limited.^3^ A 2010 policy brief on eye care equity in Nepal highlighted that gender disparity in eye care is persistent, profound, and pervasive.^4^

To better understand the barriers women faced, Tilanga Institute of Ophthalmology carried out formative research in 2016 which concluded that the cost of eye care services and the lack-of female-friendly care were the major barriers. This was supported by qualitative exit interviews with women about the specific changes that would make eye health facilities more female-friendly for them.

**Figure:**
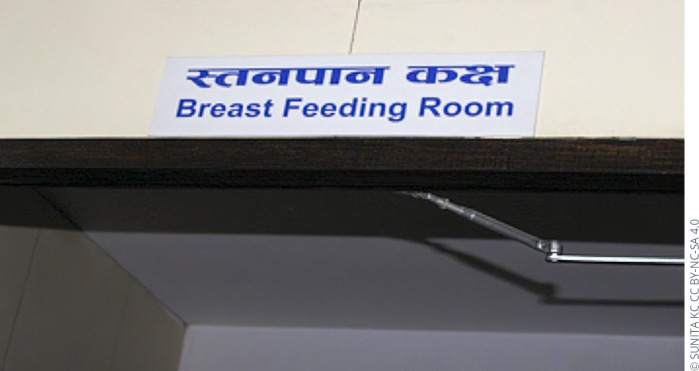
Female-friendly facilities such as an enclosed breastfeeding space can improve access to eye care for women. **NEPAL**

Based on these findings, Tilganga Institute of Ophthalmology, with support from the Fred Hollows Foundation (FHF), conducted a pragmatic trial of strategies to promote access to eye care for women in remote and marginalised areas in five districts of eastern and far-western Nepal, including the hill regions and the *terai* (lowlands). The strategies were delivered through two intervention packages, from 2018 to 2020.

The first intervention package focused on the delivery of a set of strategies that collectively sought to address the ‘awareness’ and ‘acceptability’ dimensions of access, by enhancing women’s experience of care and their awareness of services. This included:

- making eye health facilities female-friendly by setting up separate queues and toiles for men and women, as well as an enclosed breastfeeding space
- appointing a focal person to deal with gender issues within each hospital
- mobilising and training female community health volunteers to carry out eye health promotion and referral
- training technical and health personnel at eye care facilities
- hosting women-focused eye health awareness events in selected community eye care centres
- promoting eye health through mothers’ groups
- distributing information, education, and communication (IEC) materials
- reaching out through mass media campaigns on local FM radio services
- conducting outreach programmes.

The second intervention package focused on the additional benefits associated with reducing the non-medical, out-of-pocket costs associated with eye care, thereby addressing the ‘affordability’ dimension of access. This included:

- free eye treatment and free surgery for low-income and marginalised female patients referred by female community health volunteers, as well as for women referred at outreach camps in all intervention districts
- financial support for travel, food, and accommodation for the patients and for one family member accompanying each patient.

Data were collected at baseline (before the packages were implemented) and at the end of the study period, using a mixed method approach at the level of service providers and the community. The quantitative results were analysed using the difference-in-differences method, which compared the changes observed at the intervention sites with that of the control. The results were further supported by qualitative findings that were transcribed, reviewed, and analysed manually by identifying themes and categories.

After one year of the intervention, it was observed that – in the intervention sites – awareness-raising activities for women increased their knowledge about cataract. The work of female community health volunteers at the community level also led to an increase in women’s self-reported autonomy in decision-making about accessing eye health care, and women cited female community health volunteers as a preferred source of eye health information. Most importantly, travel barriers decreased after intervention, with the provision of financial support to cover the travel costs of accessing eye care. However, the interventions could not increase women’s access to cataract surgery at distant tertiary eye hospitals, with women citing household responsibilities as the main barrier. Instead, there was a surge of female service seekers in the outreach camps that were closer to their homes.

**“Awareness-raising activities for women increased their knowledge about cataract.”**

Based on the findings from the trial, the following measures could be adopted by eye health service providers to reduce the gender disparity in eye care access in other parts of Nepal and in countries with rural, marginalised populations, and where women have to depend on their male counterparts for decision-making.

## At the institutional level

- In all eye hospitals, there should be a dedicated team for gender and eye health programmes led by a gender focal person with specific terms of reference. The overall responsibilities of the focal person would be to ensure the delivery of gender-responsive services, support policies to enhance gender equality, arrange periodic training for staff on gender issues, and so on.
- There should be periodic reviews of the needs and expectations of female patients, by conducting exit interviews and assessing the uptake of clinical services (including cataract surgery) by female patients.
- Periodic review of the records of female patients (regular patients, as well as those referred by female community health volunteers and via outreach camps), including those of women from marginalised groups, can provide useful feedback to hospital management teams. Disaggregated data by gender, ethnicity, and area can be used to monitor interventions and reduce disparities in eye care access and delivery.
- Regular communication should be established with community clinics in the catchment areas to collect information regarding women’s use of eye care services, including difficulties faced while providing eye services to female patients.
- Eye care providers should provide regular updates about women’s eye health needs, expectations, issues, and challenges.

**Figure:**
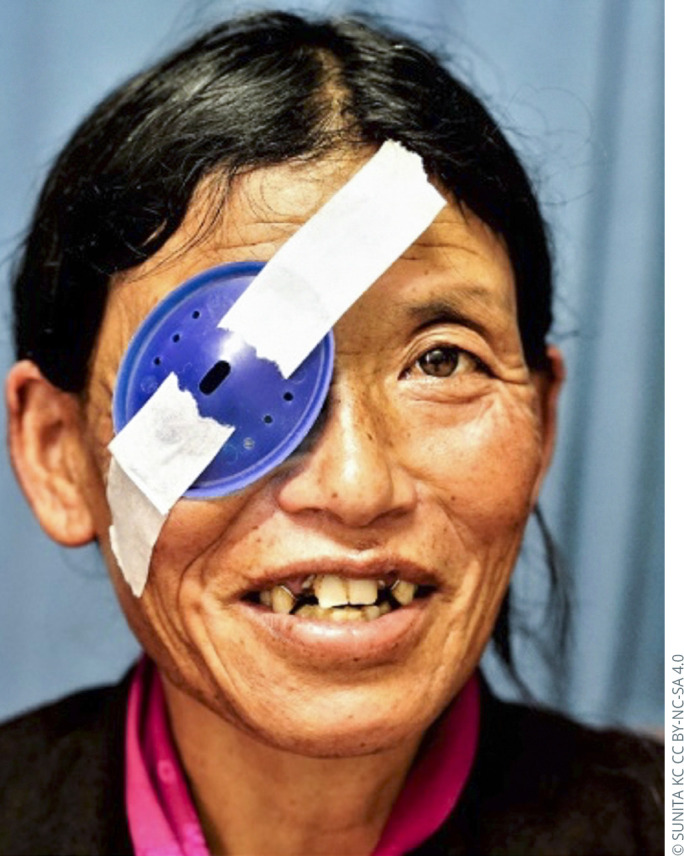
A woman after cataract surgery. **NEPAL**

## At the community level

- Regular communication should be established with local governments and local health facilities to promote community-based eye health programmes and strengthen the referral mechanism in collaboration with local female community health volunteers (where available) and other stakeholders.
- The integration of cataract referral programmes with other local health programmes, where appropriate and feasible, can reduce staff effort, increase coordination, reduce the time spent on programme activities, and possibly increase the coverage area.

## At national level

In Nepal, there is a strong structural network of primary health care centres across all the administrative units. This study showed that women could be reached and encouraged to use eye care services through the community. Thus, integrating eye care services into existing primary health care programmes will increase the availability of eye care at the grassroots level, to which women have easier access.
